# PBU Concentration-Dependent Regulation of Callus Differentiation in *Eucalyptus urophylla × E. grandis*: Integrated miRNA and Metabolomic Insights

**DOI:** 10.3390/biology15151315

**Published:** 2026-08-06

**Authors:** Chaohong Wang, Taoming Yang, Lejun Ouyang, Jiapeng Zeng, Kang Xun, Limei Li, Bingwei Jiang

**Affiliations:** 1 College of Biology and Food Engineering, Guangdong University of Petrochemical Technology, Maoming 525000, China; 2 Guangdong Provincial Engineering and Technology Research Center for Gene Resource Exploration and Precision Editing of Lingnan Characteristic Plants, Maoming 525000, China; 3 Guangdong Provincial Key Laboratory of Green and Intelligent Agricultural Production, Maoming 525000, China; 4 Maoming Agricultural Science and Technology Promotion Center, Maoming 525000, China

**Keywords:** plant regeneration, cytokinin signaling, tissue culture, organogenesis, redox regulation, miRNA–target prediction, multi-omics integration

## Abstract

PBU (N-phenyl-N′-thiazolylurea) promotes callus induction and adventitious bud differentiation in eucalyptus, but the miRNA-mediated regulatory mechanisms underlying these effects remain unclear. In this study, calli of *Eucalyptus urophylla × E. grandis* clone DH32-29 with distinct phenotypes were cultured at four PBU concentrations (0, 0.1, 1 and 5 mg L^−1^) and analyzed by small-RNA sequencing, targeted metabolomics, and qRT-PCR validation. A total of 114 common differentially expressed miRNAs were identified, targeting 610 mRNAs. Functional enrichment analysis revealed that these targets were predominantly associated with lignin metabolism, phenylpropanoid metabolism, biotin metabolism, tryptophan metabolism, protein processing in the endoplasmic reticulum, and galactose metabolism. Metabolomic profiling detected 3029 metabolites, with differential metabolites enriched in the ABC transporter pathway, galloyl sugar biosynthesis, and cofactor biosynthesis. Key miRNA families, including miR164, miR165/166, and miR396, exhibited PBU concentration-dependent expression patterns and were predicted, based on in silico target prediction and qRT-PCR co-expression, to be potentially associated with target genes involved in lignin biosynthesis, ROS-related metabolism, and cytokinin homeostasis; these regulatory relationships remain to be experimentally validated. Among the tested concentrations, 1 mg L^−1^ PBU was the dosage associated with the strongest reprogramming of secondary metabolism and with metabolic signatures suggestive of better preserved redox homeostasis; future work will build on this reference dataset with quantitative regeneration phenotyping and direct redox measurements to confirm this candidate optimum. These findings provide new insights into PBU-mediated in vitro regeneration in eucalyptus and offer a molecular basis for optimizing regeneration systems in *E. urophylla × E. grandis*. These findings provide new insights into the miRNA-metabolite regulatory network underlying phenylurea-mediated callus differentiation in woody plants.

## Simple Summary

*Eucalyptus urophylla* × Eucalyptus grandis is a major commercial hardwood tree widely propagated via tissue culture. However, unstable and inefficient callus differentiation limits its large-scale industrial breeding. The cytokinin PBU strongly promotes eucalyptus shoot regeneration in a strictly concentration-dependent manner, yet the underlying molecular mechanism remains unclear. Here, we integrated microRNA sequencing and metabolomic analysis to explore PBU-regulated callus differentiation. Multiple key microRNAs, target genes, and metabolic pathways related to hormone signaling, flavonoid biosynthesis, and amino acid metabolism were identified. This study clarifies the molecular regulatory network of PBU-mediated morphogenesis and provides valuable references for optimizing eucalyptus tissue-culture systems.

## 1. Introduction

Eucalyptus is a fast-growing, highly adaptable broad-leaved tree species widely cultivated in tropical and subtropical regions, with applications in timber production, ecological protection, and climate regulation [1,2]. However, eucalyptus is a cross-pollinated species, and sexual reproduction easily leads to high trait segregation in progeny, whereas asexual reproduction technologies face challenges in large-scale application due to low survival rates [3]. Currently, plant in vitro regeneration technologies have been widely applied in crops such as tobacco [4], rice [5], chrysanthemum [6], and tomato [7]. Meanwhile, tissue-culture technologies have been studied in approximately 60 eucalyptus species worldwide, and while most have successfully achieved in vitro plant regeneration, they generally encounter issues such as low regeneration efficiency, strong genotype dependence, and poor stability [8,9,10].

In eucalyptus in vitro regeneration systems, hormone regulation is key to callus formation and adventitious bud differentiation. The synergistic effect of auxin and cytokinin dominates callus formation, bud-primordium induction, and subsequent organogenesis [11]. Gibberellin affects xylem development by regulating cell division and secondary cell wall synthesis [12]; abscisic acid participates in stress responses and can promote the conversion of callus into embryogenic callus during somatic embryogenesis [13]; jasmonic acids activate defense networks and promote secondary metabolite accumulation [14]. PBU is a phenylurea-type cytokinin related to thidiazuron and is used to promote cell division, callus formation, and adventitious bud differentiation [15,16]. miRNAs, a class of endogenous single-stranded small-RNA molecules 20–24 nt in length, precisely regulate the spatiotemporal expression of target genes by cleaving target mRNAs or inhibiting their translation, and play important roles in embryogenic callus formation and adventitious bud development [17,18]. The functions of several highly conserved miRNA families have been revealed: for example, miR319 targets the TEOSINTE BRANCHED1/CYCLOIDEA/PROLIFERATING CELL FACTOR (TCP) family and participates in leaf morphogenesis and lignin synthesis [19,20,21,22]. miR396 targets growth-regulating factor (GRF) genes and is widely involved in regulating plant growth and in vitro regeneration [23,24,25]. Meanwhile, miR396-associated modules have been implicated in salinity adaptation [26], and the rice miR396b/GRF6 module has been shown to enhance stress adaptation by increasing reactive oxygen species (ROS)-scavenging capacity [27]. In addition, miR164–NAC and miR165/166–HD-ZIP III modules have been demonstrated to regulate organ development, stress responses, and in vitro shoot regeneration [28,29]. These studies suggest that miRNAs may be key molecular hubs connecting hormone signaling, ROS homeostasis, and cell differentiation.

Despite these advances, the molecular basis of the strictly concentration-dependent action of PBU on eucalyptus callus differentiation remains poorly understood. In particular, it is unclear which miRNA–target modules mediate the switch between differentiation-permissive and differentiation-inhibitory responses at low, moderate and high PBU doses, and how such miRNA-directed regulation is coupled to downstream metabolic reprogramming. An integrated multi-omics approach that combines small-RNA sequencing with targeted metabolomics is well suited to address this gap, because it captures both the post-transcriptional regulatory layer (miRNA–target predictions) and the resulting metabolic state (hormones, phenolics, sugars, cofactors) within the same samples; this dual-layer perspective is difficult to reach with transcriptomics or metabolomics alone, and provides a broader and more integrated framework for identifying candidate regulatory modules to be tested by future functional experiments.

The specific objectives of this study were: (i) to profile miRNA expression in *E. urophylla × E. grandis* callus induced by four PBU concentrations (0, 0.1, 1 and 5 mg L^−1^) using small-RNA sequencing; (ii) to characterize the accompanying metabolic reprogramming, including endogenous hormones, phenolics, sugars and cofactors, using targeted metabolomics; (iii) to identify candidate miRNA–target modules—with a focus on miR164, miR165/166 and miR396—potentially linking PBU dosage to lignin biosynthesis, ROS-related metabolism and cytokinin homeostasis; and (iv) to validate their expression patterns by quantitative reverse-transcription PCR (qRT-PCR). Our working hypothesis is that PBU exerts its concentration-dependent effect on eucalyptus callus differentiation, at least in part, by fine-tuning a small set of conserved miRNA–target modules that in turn shape metabolic flux, and that identifying these candidate modules will provide a molecular framework for future functional studies aimed at optimizing eucalyptus in vitro regeneration systems.

## 2. Materials and Methods

### 2.1. Material Treatment

Stem segments of sterile seedlings of *E. urophylla × E. grandis* clone DH32-29 were used as explants and cultured on 1/2 MS callus medium (2.3 g L^−1^ 1/2 MS, 30 g L^−1^ sucrose, 7 g L^−1^ agar, 100 mg L^−1^ Vc, 0.05 mg L^−1^ NAA, 500 μmol L^−1^ Put, 100 μmol L^−1^ Spd) containing 0, 0.1, 1, and 5 mg L^−1^ PBU. Six-week-old callus tissues were used as the starting material, and 1 g of callus was randomly sampled from each treatment, snap-frozen in liquid nitrogen, and divided into two equal parts: one part was used for small-RNA sequencing, and the other part was vacuum freeze-dried for 63 h, ground (30 Hz, 5 min), and stored at −80 °C for metabolomic and hormone quantification. Samples were numbered as A (0 mg L^−1^), B (0.1 mg L^−1^), C (1 mg L^−1^), and D (5 mg L^−1^).

### 2.2. RNA Extraction and sRNA Library Construction

Total RNA was extracted from frozen samples using a FastPure Universal Plant Total RNA Isolation Kit (Cat. No. RC411-01; Vazyme Biotech Co., Ltd., Nanjing, China). For each PBU concentration (0, 0.1, 1 and 5 mg L^−1^), three independent biological replicates were prepared, yielding a total of 12 sRNA libraries (4 treatments × 3 biological replicates); libraries were not pooled across replicates. Independently harvested tissue material was also used for the metabolomic and endogenous hormone analyses (*n* = 3 biological replicates per treatment). Small-RNA library construction, library quality control and sequencing were performed by Gene Denovo Biotechnology Co., Ltd. (Guangzhou, China) on an Illumina platform. 

### 2.3. miRNA Prediction and GO/KEGG Enrichment Analysis

Raw sequencing data were processed to remove adapters and filter out low-quality reads and reads containing 5′ adapters. The resulting clean reads were aligned to the GenBank database, Rfam database, ribosome database, and the *E. urophylla × E. grandis* reference genome. Unaligned clean tags were then aligned to miRNA precursor sequences in the miRBase database to identify known miRNA sequences.

The expression level of each miRNA in each sample was normalized using the transcripts per million (TPM) algorithm. miRNA-target genes were predicted by psRobot_tar in the psRobot package (v1.2). Functional enrichment analysis of target genes of DEmiRNAs was performed using the GO (Gene Ontology, http://www.geneontology.org/, accessed on 22 July 2026) database. The main metabolic pathways and signal transduction pathways involved in candidate target genes of DEmiRNAs were analyzed by KEGG (Kyoto Encyclopedia of Genes and Genomes) pathway significant enrichment. Conserved miRNAs were identified by aligning the length-filtered clean reads (18–24 nt) to mature miRNA sequences in miRBase v22.1 [30] using Bowtie with at most 2 mismatches and no gaps; a read was assigned to a conserved miRNA family if it matched a mature miRBase entry over its full length. Reads that did not align to miRBase but mapped uniquely to the *E. urophylla × E. grandis* clone DH32-29 reference genome, folded into a canonical stem-loop precursor (MFE ≤ −20 kcal mol^−1^, minimum stem length ≥ 18 nt, maximum bulge ≤ 4 nt), and were flanked by canonical Dicer-like processing sites, were classified as novel miRNA candidates using miRDeep-P2 ( [31]) with default parameters.

### 2.4. Screening of Differentially Expressed miRNAs and Prediction/Analysis of Their Target mRNAs

Differentially expressed miRNAs (DEmiRNAs) among the four treatments of *E. urophylla × E. grandis* were screened using DEGseq (v1.42.0) with TMM normalization and Benjamini–Hochberg false discovery rate (FDR) correction, applying |log2 fold change (FC)| ≥ 1 and adjusted *p* ≤ 0.05. Complementary pairing of miRNAs and target genes was performed using patmatch_v1.2 software. Putative miRNA-target genes were predicted, and the regulatory relationships were counted and visualized.

The target genes of miRNAs were predicted using psRobot software (v1.2). GO functional annotation and KEGG pathway analysis of the mRNAs targeted by DEmiRNAs were performed using the GOseqR (goseq R package v1.50.0) package and KOBS software (KOBAS v3.0, http://kobas.cbi.pku.edu.cn/, accessed on 22 July 2026). miRNA–target pairs were retained using the default parameters of psRobot (v1.2) and psRNATarget (2017 release)—namely, an expectation score ≤ 3.0 combined with the built-in minimum-free-energy cutoff recommended by the respective tools for plant miRNAs—and further filtered by *p* ≤ 0.05. The resulting regulatory network was visualized in Cytoscape (v3.9.1).

Target genes of miR165/166, miR164, and miR396 family members were predicted using the online tool RNATarget (https://www.zhaolab.org/psRNATarget/, accessed on 22 July 2026) with default parameters. Functional annotation of target genes was performed using the NCBI Nucleotide database (https://www.ncbi.nlm.nih.gov/, accessed on 22 July 2026). For every predicted miRNA target gene selected for the final result set, functional annotation was assigned by BLASTx (NCBI BLAST+ v2.13.0) alignment (E-value ≤ 1 × 10^−10^) of the target CDS against NCBI non-redundant protein and UniProtKB/Swiss-Prot databases, followed by Pfam v35.0 domain assignment (hmmscan (HMMER v3.3.2, http://hmmer.org/), E-value ≤ 1 × 10^−5^); the top annotated hit and its Pfam family were retained and are cross-checked against the family-level literature cited in the Discussion.

### 2.5. Metabolomic and Endogenous Hormone Quantification

To 50 mg of dried powder, 200 µL of 70% methanol (containing 250 µg mL^−1^ internal standard) pre-cooled to −20 °C was added, and the mixture was vortexed for 30 s six times at 4 °C, centrifuged at 12,000 rpm for 3 min, and filtered through a 0.22 µm membrane. Peak areas were extracted using Analyst software (version 1.6.3, AB SCIEX, Concord, ON, Canada), and dimensionality reduction was performed by PCA and orthogonal partial least squares discriminant analysis (OPLS-DA). Differential metabolites were screened by variable importance in projection (VIP) > 1 and *p* < 0.05, and pathways were annotated using KEGG and MetMap.

The same batch of dry powder was used to quantify endogenous plant hormones, including auxin, abscisic acid, jasmonic acid, and salicylic acid, by liquid chromatography–tandem mass spectrometry (LC-MS/MS) at Gene Denovo Biotechnology Co., Ltd. (Guangzhou, China), including IAA, ABA, JA, and SA.

### 2.6. Expression Analysis of Related Genes

qRT-PCR was used to validate the expression of the predicted target genes of miR164, miR165/166 and miR396 (i.e., members of the NAC, HD-ZIP III, GRF and cytokinin oxidase/dehydrogenase (CKX) families listed in Table 1); miRNA levels themselves were not re-measured by stem-loop qRT-PCR but were quantified directly from the sRNA-seq TPM values reported in Section 3.2. Total RNA was extracted from the same four callus tissues of *E. urophylla × E. grandis* treated with the four PBU concentrations, and relative expression levels of the target genes were determined using the Eucalyptus *Actin* gene (*EgActin*, GenBank accession XM_010038187.3, referred to as “Actin” in Table 1) as the internal reference gene, whose suitability as a candidate reference gene in eucalyptus has been systematically evaluated across organs and environmental conditions [32] and used by our group as the qRT-PCR reference gene in the same *E. urophylla × E. grandis* clone DH32-29 [33]. First-strand cDNA was synthesised from total RNA using a HiScript III RT SuperMix for qPCR (+gDNA wiper) (Cat. No. R323-01; Vazyme Biotech Co., Ltd., Nanjing, China), which removes residual genomic DNA prior to reverse transcription. Real-time qRT-PCR was performed using an Archimed X4 real-time fluorescence quantitative PCR system (Kunpeng (Xuzhou) Scientific Instruments Co., Ltd., Xuzhou, China) and the standard 20 µL reaction protocol of the ChamQ Universal SYBR qPCR Master Mix (Cat. No. Q711-02; Vazyme Biotech Co., Ltd., Nanjing, China), containing 10.0 µL of 2× ChamQ Universal SYBR qRT-PCR Master Mix, 0.4 µL each of 10 µmol L^−1^ forward and reverse primers, 1.0 µL of cDNA template (50 ng µL^−1^), and ddH_2_O to a final volume of 20.0 µL. The amplification protocol comprised an initial denaturation at 95 °C for 1 min, followed by 40 cycles of 95 °C for 10 s and 60 °C for 30 s. Relative gene expression levels were calculated using the 2^−ΔΔCt^ method, based on three biological replicates. Graphs were plotted using GraphPad Prism 7.0 (GraphPad Software LLC, San Diego, CA, USA), and primer sequences are shown in Table 1. Statistical analyses of qRT-PCR data were performed in GraphPad Prism 7.0 using one-way ANOVA followed by Dunnett’s multiple-comparison post hoc test against the control group A (0 mg L^−1^ PBU); all *p* values were adjusted for multiple testing using the Benjamini–Hochberg (FDR) procedure, and significance is reported at * *p* ≤ 0.05, ** *p* ≤ 0.01, and *** *p* ≤ 0.001. For sRNA-seq and metabolomic datasets, differential features were called with Benjamini–Hochberg FDR-adjusted *p* ≤ 0.05 and |log2FC| ≥ 1, as specified in Section 2.4 and Section 2.5. To quantitatively evaluate the strength of the predicted miRNA–target relationships, Spearman rank correlation coefficients (rho) between miRNA TPM values (sRNA-seq, n = 12 libraries) and the corresponding target-gene 2^−ΔΔCt^ values (qRT-PCR, n = 12) were computed for every miRNA–target pair; pairs with rho ≤ −0.5 and Benjamini–Hochberg FDR-adjusted *p* ≤ 0.05 were considered consistent with the predicted repressive regulation.

## 3. Results

### 3.1. Quality Control and Evaluation of Sequencing Data

In this study, sRNA-seq was performed on *E. urophylla × E. grandis* callus treated with various PBU concentrations, and a total of 12 libraries were constructed. After quality control of the raw Illumina sequencing data, the number of effective reads per library ranged from 7,436,910 to 14,019,006, with alignment rates between 93.61% and 98.52%. The Pearson correlation coefficients between biological replicates were all greater than 0.932, indicating that the sRNA-seq data were of high quality and suitable for subsequent analysis.

### 3.2. miRNA Expression, Differential Analysis, and Hierarchical Clustering Analysis

**Figure 1:**
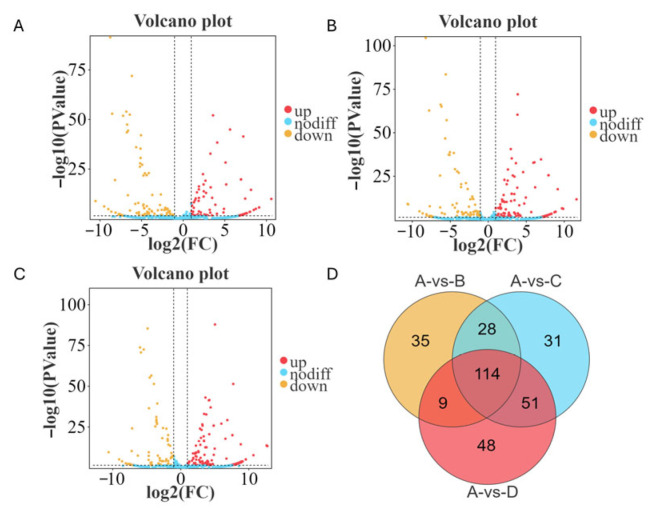
Volcano and Venn plots of differentially expressed miRNAs (DEmiRNAs) in *E. urophylla × E. grandis* callus treated with different PBU concentrations. Comparisons B vs. A, C vs. A and D vs. A correspond to 0.1, 1 and 5 mg L^−1^ PBU versus the 0 mg L^−1^ control, respectively. DEmiRNAs were identified with DEGseq using |log2FC| ≥ 1 and Benjamini–Hochberg adjusted *p* ≤ 0.05. (**A**) Volcano plot for B vs. A; (**B**) volcano plot for C vs. A; (**C**) volcano plot for D vs. A; (**D**) Venn diagram of the DEmiRNA sets from the three comparisons. Dashed lines in the volcano panels mark the |log2 FC| = 1 and adjusted-*p* = 0.05 significance thresholds.

The read counts of miRNAs were statistically analyzed and normalized using TPM. Using the criteria of |log2FC| ≥ 1 and adjusted *p* ≤ 0.05, DEGseq differential analysis identified 114 common differentially expressed miRNAs (DEmiRNAs) across the PBU treatment groups relative to the control group, targeting 610 mRNAs. The low PBU concentration (0.1 mg L^−1^) significantly upregulated miRNAs such as miR164 and miR396, whereas the high concentration (5 mg L^−1^) inhibited miR165/166 expression, indicating that PBU concentration significantly affects miRNA expression (Figure 1).

### 3.3. Functional Enrichment Analysis of miRNAs

**Figure 2:**
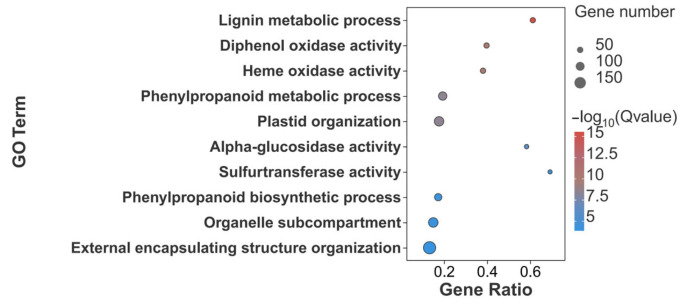
GO enrichment map of differentially expressed miRNAs in B vs. A comparison. Enrichment significance was determined by a hypergeometric test with Benjamini–Hochberg FDR correction; only GO terms with adjusted *p* ≤ 0.05 are displayed. Dot size reflects the number of target genes assigned to each term.

**Figure 3:**
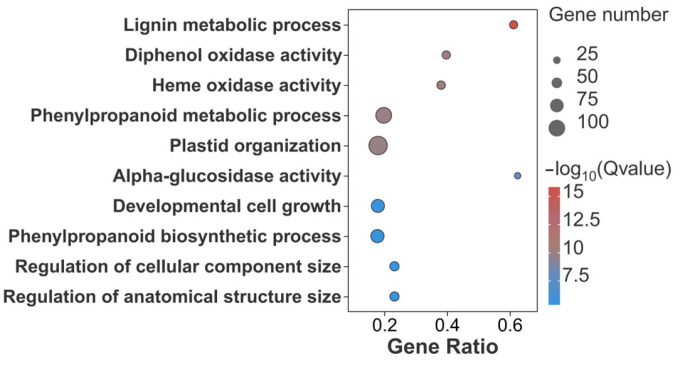
GO enrichment map of differentially expressed miRNAs in C vs. A comparison. Enrichment significance was determined by a hypergeometric test with Benjamini–Hochberg FDR correction; only GO terms with adjusted *p* ≤ 0.05 are displayed. Dot size reflects the number of target genes assigned to each term.

**Figure 4:**
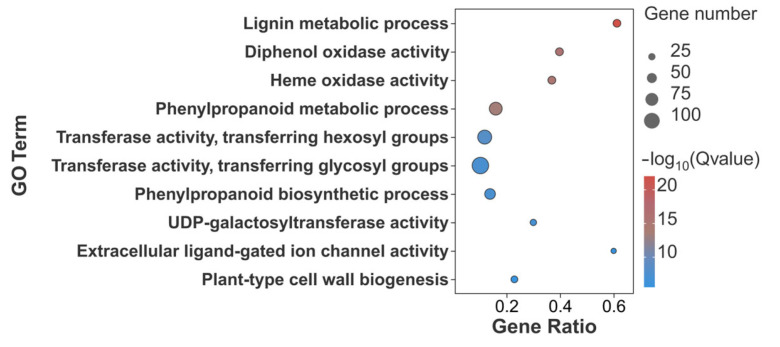
GO enrichment map of differentially expressed miRNAs in D vs. A comparison. Enrichment significance was determined by a hypergeometric test with Benjamini–Hochberg FDR correction; only GO terms with adjusted *p* ≤ 0.05 are displayed. Dot size reflects the number of target genes assigned to each term.

To further investigate the biological processes associated with the DEmiRNAs, GO and KEGG enrichment analyses were performed on the candidate target genes of these DEmiRNAs. Pathway enrichment analysis revealed that, although the enriched pathways varied somewhat among the different PBU treatments of *E. urophylla × E. grandis* callus, the most significantly correlated pathways and those with the highest impact factors were largely consistent, namely the lignin metabolic process and the phenylpropanoid metabolic process (Figure 2, Figure 3 and Figure 4).

**Figure 5:**
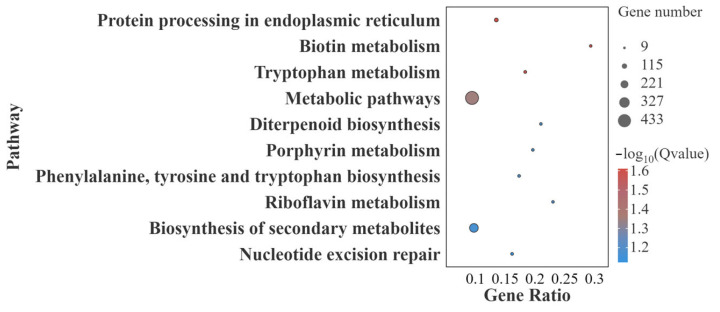
KEGG enrichment map of differentially expressed miRNAs in B vs. A comparison. KEGG pathway significance was determined by a hypergeometric test with Benjamini–Hochberg FDR correction; pathways with adjusted *p* ≤ 0.05 are shown. Dot size reflects the number of target genes assigned to each pathway.

**Figure 6:**
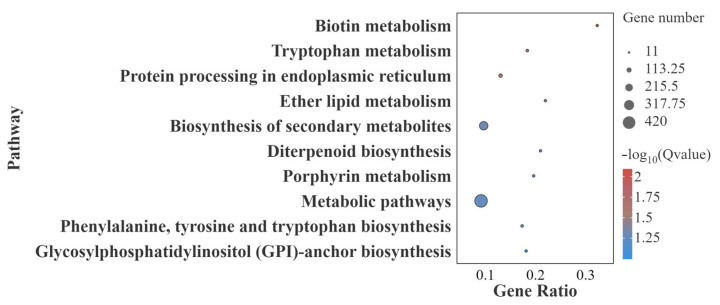
KEGG enrichment map of differentially expressed miRNAs in C vs. A comparison. KEGG pathway significance was determined by a hypergeometric test with Benjamini–Hochberg FDR correction; pathways with adjusted *p* ≤ 0.05 are shown. Dot size reflects the number of target genes assigned to each pathway.

**Figure 7:**
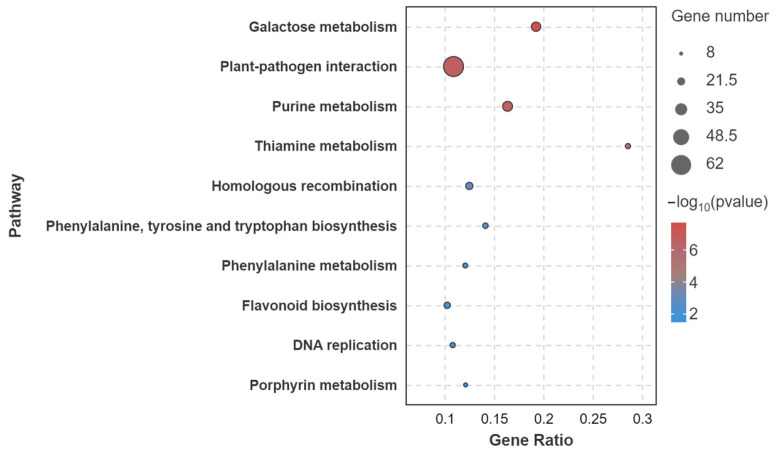
KEGG enrichment map of differentially expressed miRNAs in D vs. A comparison. KEGG pathway significance was determined by a hypergeometric test with Benjamini–Hochberg FDR correction; pathways with adjusted *p* ≤ 0.05 are shown. Dot size reflects the number of target genes assigned to each pathway.

KEGG pathway enrichment analysis showed that candidate target genes were significantly enriched in biotin metabolism, tryptophan metabolism, protein processing in the endoplasmic reticulum, and galactose metabolism. These results indicate that PBU affects metabolism and differentiation of *E. urophylla × E. grandis* callus by regulating miRNAs (Figure 5, Figure 6 and Figure 7).

### 3.4. Target-Gene Prediction and Expression Validation

**Figure 8:**
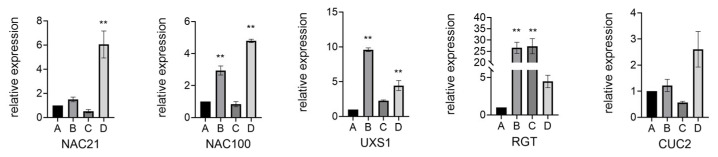
Differential expression of miR164 target genes in *E. urophylla × E. grandis* treated with different PBU concentrations. Groups A, B, C and D on the *x*-axis correspond to 0, 0.1, 1 and 5 mg L^−1^ PBU, respectively. Expression levels were normalized to *EgActin* and are shown relative to the control group A (0 mg L^−1^ PBU), which was set to 1. Data are presented as mean ± SD of three biological replicates. Asterisks indicate significant differences relative to the control group (Student’s *t*-test): ** *p* ≤ 0.01.

**Figure 9:**
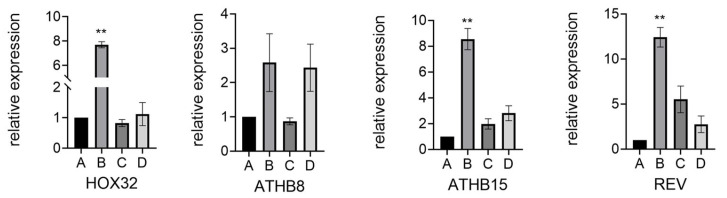
Differential expression of miR165/166 target genes in *E. urophylla × E. grandis* treated with different PBU concentrations. Groups A, B, C and D on the *x*-axis correspond to 0, 0.1, 1 and 5 mg L^−1^ PBU, respectively. Expression levels were normalized to *EgActin* and are shown relative to the control group A (0 mg L^−1^ PBU), which was set to 1. Data are presented as mean ± SD of three biological replicates. Asterisks indicate significant differences relative to the control group (Student’s *t*-test): ** *p* ≤ 0.01.

**Figure 10:**
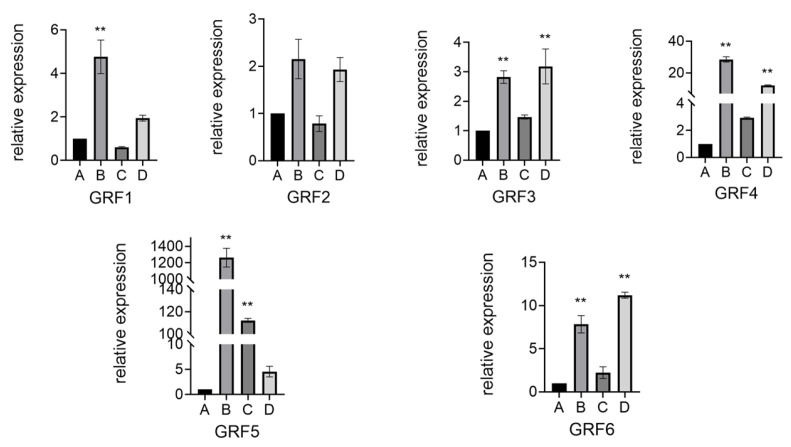
Differential expression of miR396 target genes in *E. urophylla × E. grandis* treated with different PBU concentrations. Groups A, B, C and D on the *x*-axis correspond to 0, 0.1, 1 and 5 mg L^−1^ PBU, respectively. Expression levels were normalized to *EgActin* and are shown relative to the control group A (0 mg L^−1^ PBU), which was set to 1. Data are presented as mean ± SD of three biological replicates. Asterisks indicate significant differences relative to the control group (Student’s *t*-test): ** *p* ≤ 0.01.

**Figure 11:**
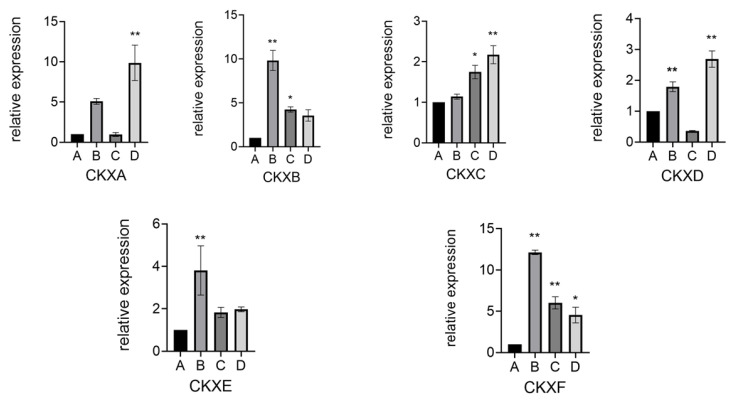
Differential expression of CKX target genes in *E. urophylla × E. grandis* treated with different PBU concentrations. Groups A, B, C and D on the *x*-axis correspond to 0, 0.1, 1 and 5 mg L^−1^ PBU, respectively. Expression levels were normalized to *EgActin* and are shown relative to the control group A (0 mg L^−1^ PBU), which was set to 1. Data are presented as mean ± SD of three biological replicates. Asterisks indicate significant differences relative to the control group (Student’s *t*-test): * *p* ≤ 0.05, ** *p* ≤ 0.01.

Among the 114 DEmiRNAs, miR164, miR165/166 and miR396 were prioritized for downstream analysis based on three explicit criteria: (i) consistent differential expression across all three PBU-treatment comparisons (B/C/D vs. A); (ii) prior functional characterization of these families in developmental and stress-related pathways in other plants; and (iii) significant enrichment of their predicted target genes in the KEGG/GO categories most strongly affected by PBU treatment in our data (lignin/phenylpropanoid metabolism, ROS-related metabolism, and cytokinin metabolism). Under these criteria, the target genes of the miR165/166, miR164, and miR396 families were predicted using the psRNATarget database (https://www.zhaolab.org/psRNATarget/, accessed on 22 July 2026). The analysis identified five target genes for miR164, four target genes for miR165/166, and six target genes for miR396 (Table 2). Target genes significantly enriched in biotin metabolism (miR165/166) and ROS metabolism (miR164, including the NAC and HD-ZIP III transcription factor families), along with the significantly differentially expressed miR396 target-gene GRFs and CKXs that promote irreversible cytokinin degradation, were selected for expression level verification by qRT-PCR to analyze the mechanism by which PBU regulates callus differentiation in *E. urophylla × E. grandis*. The qRT-PCR results are shown in Figure 8, Figure 9, Figure 10 and Figure 11.

### 3.5. Correlation Between miRNA and Target-Gene Expression

Spearman correlation between the sRNA-seq-derived miRNA TPM values and the qRT-PCR-derived relative expression of their predicted target genes across all 12 libraries revealed direction-consistent relationships for the majority of the analyzed pairs. In particular, negative correlations (rho ≤ −0.5, BH-FDR-adjusted *p* ≤ 0.05) were observed between miR164 and its canonical NAC-family targets (NAC21, NAC100, CUC2), between miR165/166 and the HD-ZIP III targets (HOX32, ATHB8, ATHB15, REV), and between miR396 and multiple GRF-family targets (GRF1-6), consistent with the predicted repressive regulation. In contrast, the unconventional miR164 predicted targets UXS1 and RGT did not show consistent negative correlations across the 12 libraries, further supporting their status as low-confidence predictions that require experimental confirmation before being interpreted as bona fide cleavage substrates.

### 3.6. Metabolomic Analysis

#### 3.6.1. Metabolite Pathway Analysis

**Figure 12:**
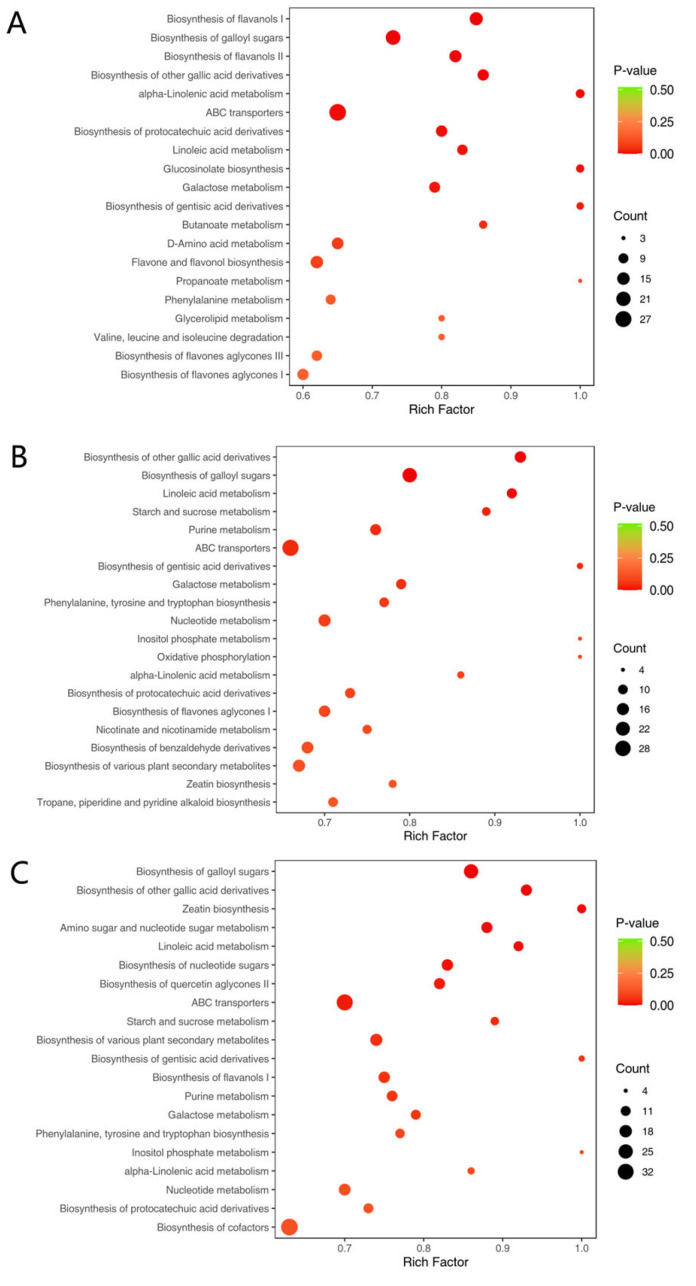
KEGG pathway enrichment of differential metabolites in *E. urophylla × E. grandis* callus treated with different PBU concentrations. Panels (**A**–**C**) show enrichment results for the B vs. A, C vs. A and D vs. A comparisons (0.1, 1 and 5 mg L^−1^ PBU vs. 0 mg L^−1^ control), respectively. Enrichment significance was assessed by a hypergeometric test with Benjamini–Hochberg FDR correction (adjusted *p* ≤ 0.05).

KEGG enrichment analysis of *E. urophylla × E. grandis* callus proliferation revealed that differential metabolites were mainly enriched in pathways such as ABC transporters, galloyl sugar biosynthesis, and cofactor biosynthesis. These pathways play important roles in plant growth, development, and stress response (Figure 12). Among the 3029 detected metabolites, differential metabolites (BH-FDR-adjusted *p* ≤ 0.05 and |log2FC| ≥ 1 vs. group A) driving the separation among PBU treatments were dominated by phenolic sugars, polyamines and cofactors. Metabolites showing the strongest positive log2FC (up-accumulation) at 1 mg L^−1^ PBU included gallic acid, galloyl acetylglucoside, 4-O-galloyl arbutin, spermidine, NAD^+^, FMN, riboflavin, and nicotinamide, all of which have well-documented roles in secondary metabolism and cofactor-dependent antioxidant/redox reactions. Metabolites showing the strongest negative log2FC (down-accumulation) at 1 mg L^−1^ PBU relative to lower concentrations included putrescine and ornithine (consistent with an activated polyamine-conversion flux toward spermidine), several free amino acids and simple sugars (consistent with mobilization into secondary metabolism). At 5 mg L^−1^ PBU, vitamin C (ascorbate) was significantly decreased whereas GA_4_ and IAA were significantly increased. These metabolic signatures are interpreted as pathway-level indicators of the physiological state of the callus rather than as direct proof of any specific redox mechanism, which would require targeted quantification of H_2_O_2_, $O_{2}^{•-}$, MDA, and antioxidant-enzyme activities.

#### 3.6.2. Endogenous Hormone Determination

**Figure 13:**
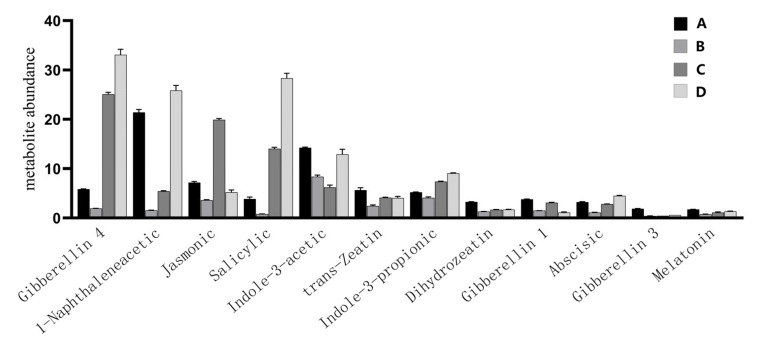
Changes in endogenous hormone contents under different PBU concentrations. Groups A, B, C and D correspond to 0, 0.1, 1 and 5 mg L^−1^ PBU, respectively. Bar colours denote the four PBU treatments: A (0 mg L^−1^), B (0.1 mg L^−1^), C (1 mg L^−1^), D (5 mg L^−1^). Statistical significance versus the control group A was evaluated by Student’s *t*-test. *X*-axis category labels are rotated 45° for readability. Error bars represent mean ± SD of three biological replicates. 1-Naphthaleneacetic acid (NAA) is annotated as ‘exogenous (residual from medium, 0.05 mg L^−1^)’ and is not an endogenous hormone; it is retained solely to show inter-treatment accumulation.

The results showed that the contents of plant hormones in *E. urophylla × E. grandis* callus varied significantly under different PBU concentrations. Compared with the control group, the contents of gibberellin 4 (GA4), 1-naphthaleneacetic acid (NAA), salicylic acid (SA), indolepropionic acid (IPA), and abscisic acid (ABA) in the callus gradually increased with increasing PBU concentration. Jasmonic acid (JA) showed the highest abundance at 1 mg L^−1^ PBU. The abundance of indole-3-acetic acid (IAA) decreased with increasing PBU concentration, but at 5 mg L^−1^ PBU it increased to a level comparable to that in the control group (Figure 13). It should be noted that 1-naphthaleneacetic acid (NAA) is a synthetic auxin analog that is not biosynthesized by plants; its detection in the callus reflects residual uptake from the culture medium (0.05 mg L^−1^ NAA, see Section 2.1) rather than endogenous biosynthesis. NAA was therefore retained in Figure 13 solely as an indicator of differential tissue accumulation among treatments and was excluded from the downstream interpretation of endogenous hormone dynamics, which instead focused on the endogenous auxin indole-3-acetic acid (IAA).

## 4. Discussion

In this study, sRNA-seq was used to analyze the miRNA expression profiles of *E. urophylla × E. grandis* callus induced by different concentrations of PBU, and a total of 1063 miRNAs were identified. The miRNAs were mainly 18–24 nt in length, with a preference for U at the first base, consistent with established plant small-RNA characteristics [34]. MicroRNAs guide sequence-specific transcript cleavage or translational repression, thereby exerting broad post-transcriptional control over gene-expression networks [35]. The analysis identified 114 commonly expressed DEmiRNAs, including miR164 and miR396 family members that have been implicated in ROS-related stress responses in other plant species [27,29]. Notably, the expression of miR396 was upregulated with increasing PBU concentration, while miR165/166 expression was downregulated, indicating that they may mediate the concentration-dependent effect of PBU. Based on these expression patterns and by analogy with regulatory modules previously described in other species, we propose the following working model, which should be regarded as a set of hypotheses to be tested by future functional experiments rather than as directly demonstrated causal relationships in eucalyptus. Under low PBU concentrations, reduced miR165/166 expression may relieve HD-ZIP III silencing and could contribute to activation of the REV-ARF-ATHB8/15 module previously implicated in bud-primordium polarity and vascular differentiation [36,37]; a moderate level of miR164 may allow partial NAC activity, which has been linked to H_2_O_2_-related signaling in previous reports [29,38]; and reduced miR396 could release GRF activity, which has been associated with enhanced callus regeneration in alfalfa [39]. Consistent with this framework, the expression of CKXA/C/D in our data was relatively low under favorable PBU dosage, which—by analogy with maize/Arabidopsis—would be expected to preserve endogenous cytokinin activity [40]. Under high PBU (5 mg L^−1^), the observed strong inhibition of miR164, upregulation of miR396, and induction of CKXA/C/D would be predicted, under this model, to disturb the NAC-ROS-related network, dampen GRF activity, and accelerate cytokinin turnover, thereby disfavoring differentiation [16,27]. We emphasize that this model is inferential: it does not include direct measurements of endogenous cytokinin metabolites, ROS/H_2_O_2_ accumulation, antioxidant-enzyme activities, lignin content, or functional perturbation of the candidate miRNAs, all of which are explicitly listed as priorities for future work.

Beyond miRNA-directed regulation, secondary cell wall biosynthesis emerged as a prominent enrichment category among DEmiRNA targets, prompting comparison with recent progress on IRX-family regulators in other species. In cotton, *GhIRX15* is upregulated upon overexpression of *GhFSN1*/*GhMYB7* and downregulated upon downregulation of *GhGT47B*, demonstrating the decisive role of IRXs in secondary wall thickness and fiber length [41]. Phenylpropanoid metabolism, central to plant secondary metabolism, encompassed a total of 91 target genes, focused on two enzyme types: CYPs and SAMs. CYPs (cytochrome P450s) modify lignin precursors through hydroxylation; for example, *CYP84A1* converts coniferyl alcohol to 5-hydroxyconiferyl alcohol, which is further converted to sinapyl alcohol, thereby improving the antioxidant capacity and mechanical strength of lignin [42]. Consistent with the involvement of miRNA-controlled laccases in lignin biosynthesis, miR397b represses *MhLAC7* to alter lignin deposition and biotic-stress responses in apple [43]. Meanwhile, CYPs participate in IAA synthesis and signal transduction, affecting plant growth and stress responses. SAMs provide S-adenosylmethionine as a methyl donor, contributing to the methylation of monomers such as coniferyl alcohol and sinapyl alcohol and influencing the structure and function of lignin. In Arabidopsis, miR858a inhibits vascular *MYB63*/*MYB20* under ABA induction, affecting secondary wall formation via downstream CYPs/SAMs steps [44]. The galactose metabolism pathway was enriched with 26 GOLS (galactinol synthase) genes, which catalyze the synthesis of raffinose family oligosaccharides and modulate the osmotic regulation capacity of callus tissue. In other plants, miR397 has been reported to negatively regulate laccase-mediated lignin biosynthesis, while miR398 targets copper/zinc superoxide dismutases in oxidative-stress responses; whether these miRNAs contribute to the PBU-induced modulation of GOLS/raffinose metabolism in *E. urophylla × E. grandis* remains to be experimentally tested in future studies.

Metabolite abundance in *E. urophylla × E. grandis* callus exhibited regular changes across the PBU concentration gradient. At 0.1 mg L^−1^, levels of intracellular amino acids, sugars, putrescine, and ornithine were relatively high; the polyamine synthesis pathway was not fully activated, and energy consumption remained low. At 1 mg L^−1^, putrescine and ornithine decreased significantly, whereas spermidine accumulated markedly. The levels of galloyl acetylglucoside, 4-O-galloyl arbutin, and gallic acid increased, accompanied by synchronous rises in NAD^+^, FMN, riboflavin, and nicotinamide. The ABC transporter pathway was active, and the accumulation profile of gallic-acid-derived phenolics, riboflavin and NAD^+^/FMN cofactors is consistent with an enhanced ROS-scavenging capacity, although direct measurements of H_2_O_2_ and antioxidant-enzyme activities were not performed and are required to confirm this inference. At 5 mg L^−1^, synthesis of these phenolic sugars and cofactors was inhibited; vitamin C declined, while GA_4_ and IAA increased significantly. Tryptophan was directed toward the IAA pathway, and cells displayed metabolic profiles consistent with high proliferation but with metabolic signatures suggestive of oxidative imbalance, pending direct physiological confirmation. Based on these transcriptomic and metabolomic signatures, among the four tested PBU concentrations, 1 mg L^−1^ was associated with the strongest metabolic reprogramming toward secondary metabolism and with metabolic signatures suggestive of better preserved redox homeostasis, whereas a lower concentration produced smaller shifts and a higher concentration was associated with metabolic patterns suggestive of oxidative imbalance. This omics-based interpretation still requires validation by phenotypic indices in future work.

A cross-species comparison places our findings in a broader context. In Arabidopsis, the miR165/166–HD-ZIP III module patterns vascular polarity and cambial activity [36,37]; in poplar, miR319 rewires TCP-dependent leaf morphogenesis and lignin deposition [20,21]; in rice, the miR396b/GRF6 module enhances salt tolerance while reducing H_2_O_2_ accumulation and increasing ROS-scavenging enzyme activities [27]; and in castor and cotton, miR396b-GRF and IRX15 respectively control seed size and secondary wall thickening [41,45]. Against this background, the PBU-dose-dependent responses of miR164/NAC, miR165/166/HD-ZIP III and miR396/GRF observed here highlight a convergent post-transcriptional hub through which cytokinin-family signals could couple developmental competence to secondary metabolism. Importantly, the enrichment of lignin/phenylpropanoid (*CYP84A1*, SAM), cofactor (NAD^+^/FMN/riboflavin), polyamine (putrescine → spermidine) and cytokinin-turnover (CKX) pathways within the same PBU dosage window supports the view that these pathways are not operating in isolation but are crosstalking: HD-ZIP III derepression could feed vascular MYB/CYP-mediated lignification, while GRF activity coordinates redox-cofactor supply and cytokinin homeostasis. Direct functional perturbation of these modules together with quantification of endogenous cytokinin, H_2_O_2_ and lignin content will be required to test which of these cross-pathway links are causal in *E. urophylla × E. grandis* regeneration.

Taken together, this study provides the first integrated miRNA and metabolomic dataset for PBU-induced callus differentiation in *Eucalyptus urophylla × E. grandis*, and it advances the field in three concrete ways. First, by profiling four PBU concentrations across 12 non-pooled sRNA libraries and a paired targeted metabolomic panel, we establish a reference dataset—1063 identified miRNAs (conserved and novel), 114 differentially expressed miRNAs, 610 predicted targets, and 3029 metabolites—that will be a useful resource for the eucalyptus tissue-culture community. Second, we identify three conserved miRNA modules (miR164/NAC, miR165/166/HD-ZIP III and miR396/GRF) whose PBU-dose-dependent expression patterns are quantitatively supported by Spearman correlation with qRT-PCR-measured target expression, providing a well-defined short list of candidate regulators for future functional dissection. Third, we resolve a PBU concentration effect at the metabolic level, showing that 1 mg L^−1^ elicits the strongest reprogramming toward phenolic sugars, spermidine and redox-related cofactors, thereby providing a testable working model for optimizing eucalyptus regeneration protocols.

## 5. Conclusions

This study delivers the first integrated miRNA and metabolomic reference dataset for PBU-induced callus differentiation in *Eucalyptus urophylla × E. grandis*, encompassing 1063 identified miRNAs, 114 differentially expressed miRNAs, 610 predicted target genes and 3029 metabolites across four PBU concentrations. From this dataset, we resolve three PBU-dose-dependent miRNA modules—miR164/NAC, miR165/166/HD-ZIP III and miR396/GRF—whose expression patterns and target-gene correlations delineate a coherent, testable working model in which PBU dosage shapes callus fate through coordinated regulation of lignin/phenylpropanoid biosynthesis, ROS-related metabolism and cytokinin homeostasis. Among the tested concentrations, 1 mg L^−1^ PBU is associated with the strongest reprogramming toward secondary metabolism and with metabolic patterns suggestive of better preserved redox homeostasis, providing a data-supported candidate optimum for eucalyptus in vitro regeneration. Together, these findings offer a concrete miRNA–target catalog and a metabolic reference profile for the eucalyptus tissue-culture community, and set up the natural next steps of functional validation of the highlighted miRNA–target modules and quantitative regeneration phenotyping, building directly on the framework established here to further optimize cytokinin-mediated in vitro regeneration in eucalyptus.

## Figures and Tables

**Table 1:** qRT-PCR primers used in this study.

Gene	Forward Primer (5′→3′)	Reverse Primer (5′→3′)
*Actin*	TTGTGCTCAGTGGTGGAACC	TCTGCCTTTGCGATCCACAT
*GRF1*	TAACCACCCAAACCATCCTC	AACCTCGCCCACATCAGCAA
*GRF2*	GGTCGCTCCAGGATTCTGACAACT	GCGTCCCTGTCGTTCGTGCT
*GRF3*	GCACCTGCCGACCTCTTATC	TTGTTTCTGCTGCTTTGACG
*GRF4*	CCTCCGAGCCTGTCTATGTT	AGTTTCGGGAGTCTAACAAGGA
*GRF5*	GGGATTCATTGATGCCTGGTC	ATTATTGCCACCCACGGACA
*GRF6*	GGCTCTGCCTCTGCTGATTC	CTTGCCACTGTGCTGCTGTAA
*CKXA*	TGGCAAGAGTTCGACCTTCAA	CCCCATCAATCTTTGAATTCATGC
*CKXB*	TGTCTGCTGTCATACCAGATGAA	GGTTGAAGATCCTCTGCCCA
*CKXC*	ATGGAGGAGGTTCCGTCAGA	TGGATCTATTCACTAGCGTCCG
*CKXD*	CCACATTTTGGCAGTGAACGA	ACCCAGGTAAGATGGTGCAA
*CKXE*	AGTGGGTTTGAAGACTGGCA	GGTTGAAGATCCTCTGTCCAGG
*CKXF*	AGTGGGTTTGAAGACTGGCA	AACCAGGTAAGATGGTGCAA
*NAC21*	CAAAGGCAGAAAGACCG	CTTGAAGAACACCCGACA
*NAC100*	GGGTCATTTGCAGGGTC	AAGCAGGGCACGTAAGC
*UXS1*	TGATGGTCGTGTTGTGAG	TGTGATGTCTGGCTTCCT
*RGT*	CGCTACTCGTGCTCTG	GACCGCATTTCCTACA
*CUC2*	GAGCCTGAGGTCCTTGCA	GAGCCCATTCCCATTCG
*HOX32*	GTTATCTCATCCGACCTTGT	CCTCCATACTGAATCTCCC
*ATHB8*	TCAGCACCATTTGACTCCT	GCAATCACGATACCACGAC
*ATHB15*	GCACATTGGACCTTGCCTCTTCTCT	CCGAGGATATTATACTACGC
*REV*	ATCAAATCCTGCTAATCCC	CCCTCAGAAACCGAACC

**Table 2:** Predicted target genes of miR164, miR165/166 and miR396 families in *E. urophylla × E. grandis*.

Regulatory miRNA	Target Gene	Genomic Location
miR165/166	*HOX32*	Chr4:3897362–3904972
miR165/166	*ATHB8*	Chr3:6473879–6479973
miR165/166	*ATHB15*	Chr6:42133339–42140725
miR165/166	*REV*	Chr2:41588500–41595380
miR164	*NAC100*	Chr2:6750464–6752465
miR164	*NAC21*	Chr6:17235502–17239978
miR164	*UXS1*	Chr10:753837–759217
miR164	*RGT*	Chr6:4365120–4369546
miR164	*CUC2*	Chr6:13758516–13760574
miR396	*GRF1*	Chr2:40272964–40277449
miR396	*GRF2*	Chr5:16866727–16867198
miR396	*GRF3*	Chr5:61102835–61103567
miR396	*GRF4*	Chr8:36301003–36301438
miR396	*GRF5*	Chr10:37206847–37208174
miR396	*GRF6*	Chr11:31462657–31463128

Note: Genomic coordinates are based on the *E. urophylla × E. grandis* clone DH32-29 reference genome assembly [3] and are reported in the standardized format Chr<n>:start-end.

## Data Availability

The raw small-RNA sequencing data have been deposited in the NCBI Sequence Read Archive (BioProject accession pending; SRA IDs will be released upon publication). The metabolomic raw data have been submitted to the MetaboLights repository (study accession pending). The miRNA–target correlation matrix, the full list of differential metabolites, and the conserved/novel miRNA inventory are available from the corresponding author upon reasonable request and will be provided in the second-round revision.

## References

[1] Navarro M., Ayax C., Martinez Y., Laur J., El Kayal W., Marque C., Teulieres C. (2011). Two *EguCBF1* genes overexpressed in Eucalyptus display a different impact on stress tolerance and plant development. Plant Biotechnol. J..

[2] Liu T., Xie Y.J. (2020). Analysis and prospect of the driving forces for the rapid development of Eucalyptus plantations in China. Eucalypt Sci. Technol..

[3] Shen C., Li L.M., Ouyang L.J., Wang J., Xu C., Chen H., Jiang B., Wu W., Zeng J., Zeng B. (2023). High-quality genome of *E. urophylla × E. grandis* and comparative genomics provide insights on evolution and diversification of Eucalyptus. BMC Genom..

[4] Horsch R.B., Fry J.E., Hoffmann N.L., Eichholtz D., Rogers S.G., Fraley R.T. (1985). A simple and general method for transferring genes into plants. Science.

[5] Toki S., Hara N., Ono K., Onodera H., Tagiri A., Oka S., Tanaka H. (2006). Early infection of scutellum tissue with Agrobacterium allows high-speed transformation of rice. Plant J..

[6] Silva T., Jaime A., Shinoyama H., Fukumoto F., Kanno A. (2013). Chrysanthemum biotechnology: Quo vadis?. Crit. Rev. Plant Sci..

[7] McCormick S., Niedermeyer J., Fry J., Barnason A., Horsch R., Fraley R. (1986). Leaf disc transformation of cultivated tomato (*L. esculentum*) using Agrobacterium tumefaciens. Plant Cell Rep..

[8] Xie Y.J. (2000). Research progress in Eucalyptus tissue culture. World For. Res..

[9] Tan D.G., Zhuang N.S., Huang H.S. (2005). Callus induction and construction of rapid propagation system for regenerated plants of Eucalyptus 12ABL. Chin. J. Trop. Crops.

[10] Ouyang L.J., Sha Y.E., Huang Z.C., Li L.M., Xu K.J. (2012). Establishment of high-efficiency tissue culture regeneration system for Eucalyptus ‘Guangzhou No. 1’. J. Northeast For. Univ..

[11] Zhai N., Pan X., Zeng M., Xu L. (2023). Developmental trajectory of pluripotent stem cell establishment in Arabidopsis callus guided by a quiescent center-related gene network. Development.

[12] Guo G.S., Zeng B.S., Fan C.J., Qiu Z.F. (2018). Effects of exogenous GA3 application on xylem development in Eucalyptus grandis. Mol. Plant Breed..

[13] Chen Y., Yu H., Wang Y., Li F., Xing Y., Ge X. (2022). Uniconazole augments abscisic acid in promoting somatic embryogenesis in cotton (*Gossypium hirsutum* L.). Front. Plant Sci..

[14] Henery M.L., Wallis I.R., Stone C., Foley W.J. (2008). Methyl jasmonate does not induce changes in Eucalyptus grandis leaves that alter the effect of constitutive defences on larvae of a specialist herbivore. Oecologia.

[15] Guo B., Abbasi B.H., Zeb A., Xu L.L., Wei Y.H. (2011). Thidiazuron: A multi-dimensional plant growth regulator. Afr. J. Biotechnol..

[16] Liu Y.M., Wu Y.P., Li L.M., Su M., Yu Z., Ouyang L.J. (2022). Differential expression of miRNA396 and CKX genes in callus of *Eucalyptus urophylla × E. grandis* induced by different concentrations of the novel Mitogen PBU. Bull. Bot. Res..

[17] Jones-Rhoades M.W., Bartel D.P., Bartel B. (2006). MicroRNAs and their regulatory roles in plants. Annu. Rev. Plant Biol..

[18] Zhang T.Q., Lian H., Tang H., Dolezal K., Zhou C.M., Yu S., Chen J.H., Chen Q., Liu H., Ljung K. (2015). An intrinsic microRNA timer regulates progressive decline in shoot regenerative capacity in plants. Plant Cell.

[19] Chen Z.H., Qiao Z.S., Li J.Q., Zhang X.L., Ma S.J., He C.Y., Zong D. (2024). Genome-wide identification and analysis of TCP gene family in Populus yunnanensis. Biotechnol. Bull..

[20] Palatnik J.F., Allen E., Wu X., Schommer C., Schwab R., Carrington J.C., Weigel D. (2003). Control of leaf morphogenesis by microRNAs. Nature.

[21] Cheng Y., Wang L., Abbas M., Huang X., Wang Q., Wu A., Wei H., Peng S., Dai X., Li Q. (2021). MicroRNA319-mediated gene regulatory network impacts leaf development and morphogenesis in poplar. For. Res..

[22] Han P., Jing C., Han C., Xu H., Wang Y., Sun Y., Xu X., Wang S., Ni Z. (2024). Coordination of miR319-*TaPCF8* with *TaSPL14* orchestrates auxin signaling and biosynthesis to regulate plant height in common wheat. J. Integr. Plant Biol..

[23] Cao D.Y. (2018). Functional Study of miR396 in Tomato Fruit Growth and Development. Master’s Thesis.

[24] Jeong H., Kim J.H. (2019). Biological roles and an evolutionary sketch of the GRF-GIF transcriptional complex in plants. BMB Rep..

[25] Debernardi J.M., Tricoli D.M., Ercoli M.F., Hayta S., Ronald P., Palatnik J.F., Dubcovsky J. (2020). A GRF–GIF chimeric protein improves the regeneration efficiency of transgenic plants. Nat. Biotechnol..

[26] Chen X., Jiang X., Sun X., Hu Z., Gao F., Wang X., Zhang H., Chen R., Jiang Q. (2024). Gene editing and overexpression of soybean miR396a reveals its role in salinity tolerance and development. Crop J..

[27] Yuan H., Cheng M., Wang R., Wang Z., Fan F., Wang W., Si F., Gao F., Li S. (2024). miR396b/GRF6 module contributes to salt tolerance in rice. Plant Biotechnol. J..

[28] Xue T., Dai X., Wang R., Wang J., Liu Z., Xiang F. (2017). ARGONAUTE10 inhibits in vitro shoot regeneration via repression of miR165/166 in Arabidopsis thaliana. Plant Cell Physiol..

[29] Peng X., Feng C., Wang Y.T., Zhang X., Wang Y.Y., Sun Y.T., Xiao Y.Q., Zhai Z.F., Zhou X., Du B.Y. (2022). miR164g-*MsNAC022* acts as a novel module mediating drought response by transcriptional regulation of reactive oxygen species scavenging systems in apple. Hortic. Res..

[30] Kozomara A., Birgaoanu M., Griffiths-Jones S. (2019). miRBase: From microRNA sequences to function. Nucleic Acids Res..

[31] Kuang Z., Wang Y., Li L., Yang X. (2019). miRDeep-P2: Accurate and fast analysis of the microRNA transcriptome in plants. Bioinformatics.

[32] Cassan-Wang H., Soler M., Yu H., Camargo E.L.O., Carocha V., Ladouce N., Savelli B., Paiva J.A.P., Leplé J.-C., Grima-Pettenati J. (2012). Reference genes for high-throughput quantitative reverse transcription-PCR analysis of gene expression in organs and tissues of Eucalyptus grown in various environmental conditions. Plant Cell Physiol..

[33] Su M., Wu X., Wang Z., Li L., Ouyang L. (2025). Construction of an editing system for forest tree genomes based on an efficient visual screening marker in *Eucalyptus urophylla* × *Eucalyptus grandis*. Horticulturae.

[34] Axtell M.J. (2013). Classification and comparison of small RNAs from plants. Annu. Rev. Plant Biol..

[35] He L., Hannon G.J. (2004). MicroRNAs: Small RNAs with a big role in gene regulation. Nat. Rev. Genet..

[36] Donner T.J., Sherr I., Scarpella E. (2010). Auxin signal transduction in Arabidopsis vein formation. Plant Signal. Behav..

[37] Baima S., Forte V., Possenti M., Peñalosa A., Leoni G., Salvi S., Felici B., Ruberti I., Morelli G. (2014). Negative feedback regulation of auxin signaling by ATHB8/ACL5-BUD2 transcription module. Mol. Plant.

[38] Wu A., Allu A.D., Garapati P., Siddiqui H., Dortay H., Zanor M.I., Asensi-Fabado M.A., Munné-Bosch S., Antonio C., Tohge T. (2012). JUNGBRUNNEN1, a reactive oxygen species-responsive NAC transcription factor, regulates longevity in Arabidopsis. Plant Cell.

[39] Chen X.R., Yan J.P., Qiu R.M., Liu Y.R., Zhang W.J. (2022). miR396–*MsGRFs* regulate the regeneration of alfalfa callus. Acta Agrestia Sin..

[40] Morris R.O., Bilyeu K.D., Laskey J.G., Cheikh N.N. (1999). Isolation of a gene encoding a glycosylated cytokinin oxidase from maize. Biochem. Biophys. Res. Commun..

[41] Li M., Chen F., Luo J., Zhang F., Sun Z., Zhang Z., Zhao J., Wang Q., Lin M., Shi Y. (2024). The DUF579 proteins *GhIRX15s* regulate cotton fiber development by interacting with proteins involved in xylan synthesis. Crop J..

[42] Meyer K., Cusumano J.C., Somerville C., Chapple C. (1996). Ferulate-5-hydroxylase from Arabidopsis thaliana defines a new family of cytochrome P450-dependent monooxygenases. Proc. Natl. Acad. Sci. USA.

[43] Yu X., Gong H., Cao L., Hou Y., Qu S. (2020). MicroRNA397b negatively regulates resistance of Malus hupehensis to Botryosphaeria dothidea by modulating *MhLAC7* involved in lignin biosynthesis. Plant Sci..

[44] Gao Z., Su Y., Jiao G., Yin X., Wang D., Wei T., Zhu S., Zhang X., Yang X. (2025). Cell-type specific miRNA regulatory network responses to ABA stress revealed by time series transcriptional atlases in Arabidopsis. Adv. Sci..

[45] Wang X., Song Y., Li B., Yang L., Wu W., Li R., Jiang S., Fu X., Chen F., Yang D. (2024). A microRNA396b-growth regulating factor module controls castor seed size by mediating auxin synthesis. Plant Physiol..

